# Ecology and Transmission of Buruli Ulcer Disease: A Systematic Review

**DOI:** 10.1371/journal.pntd.0000911

**Published:** 2010-12-14

**Authors:** Richard W. Merritt, Edward D. Walker, Pamela L. C. Small, John R. Wallace, Paul D. R. Johnson, M. Eric Benbow, Daniel A. Boakye

**Affiliations:** 1 Department of Entomology, Michigan State University, East Lansing, Michigan, United States of America; 2 Department of Entomology and Microbiology and Molecular Genetics, Michigan State University, East Lansing, Michigan, United States of America; 3 Department of Microbiology, University of Tennessee, Knoxville, Tennessee, United States of America; 4 Department of Biology, Millersville University, Millersville, Pennsylvania, United States of America; 5 Austin Health, Melbourne, Australia; 6 Department of Biology, University of Dayton, Dayton, Ohio, United States of America; 7 University of Ghana, East Legon, Ghana; Kwame Nkrumah University of Science and Technology (KNUST) School of Medical Sciences, Ghana

## Abstract

Buruli ulcer is a neglected emerging disease that has recently been reported in some countries as the second most frequent mycobacterial disease in humans after tuberculosis. Cases have been reported from at least 32 countries in Africa (mainly west), Australia, Southeast Asia, China, Central and South America, and the Western Pacific. Large lesions often result in scarring, contractual deformities, amputations, and disabilities, and in Africa, most cases of the disease occur in children between the ages of 4–15 years. This environmental mycobacterium, *Mycobacterium ulcerans*, is found in communities associated with rivers, swamps, wetlands, and human-linked changes in the aquatic environment, particularly those created as a result of environmental disturbance such as deforestation, dam construction, and agriculture. Buruli ulcer disease is often referred to as the “mysterious disease” because the mode of transmission remains unclear, although several hypotheses have been proposed. The above review reveals that various routes of transmission may occur, varying amongst epidemiological setting and geographic region, and that there may be some role for living agents as reservoirs and as vectors of *M. ulcerans*, in particular aquatic insects, adult mosquitoes or other biting arthropods. We discuss traditional and non-traditional methods for indicting the roles of living agents as biologically significant reservoirs and/or vectors of pathogens, and suggest an intellectual framework for establishing criteria for transmission. The application of these criteria to the transmission of *M. ulcerans* presents a significant challenge.

## Author Summary

Buruli ulcer (BU) is a serious necrotizing cutaneous infection caused by *Mycobacterium ulcerans*. It is a neglected emerging disease that has recently been reported in some countries as the second most frequent mycobacterial disease in humans after tuberculosis (TB). Cases have been reported from at least 32 countries in Africa (mainly west), Australia, Southeast Asia, China, Central and South America, and the Western Pacific. BU is a disease found in rural areas located near wetlands (ponds, swamps, marshes, impoundments, backwaters) and slow-moving rivers, especially in areas prone to human-made disturbance and flooding. Despite considerable research on this disease in recent years, the mode of transmission remains unclear, although several hypotheses have been proposed. In this article we review the current state of knowledge on the ecology and transmission of *M. ulcerans* in Africa and Australia, discuss traditional and non-traditional methods for investigating transmission, and suggest an intellectual framework for establishing criteria for transmission.

## Introduction

**Figure 1:**
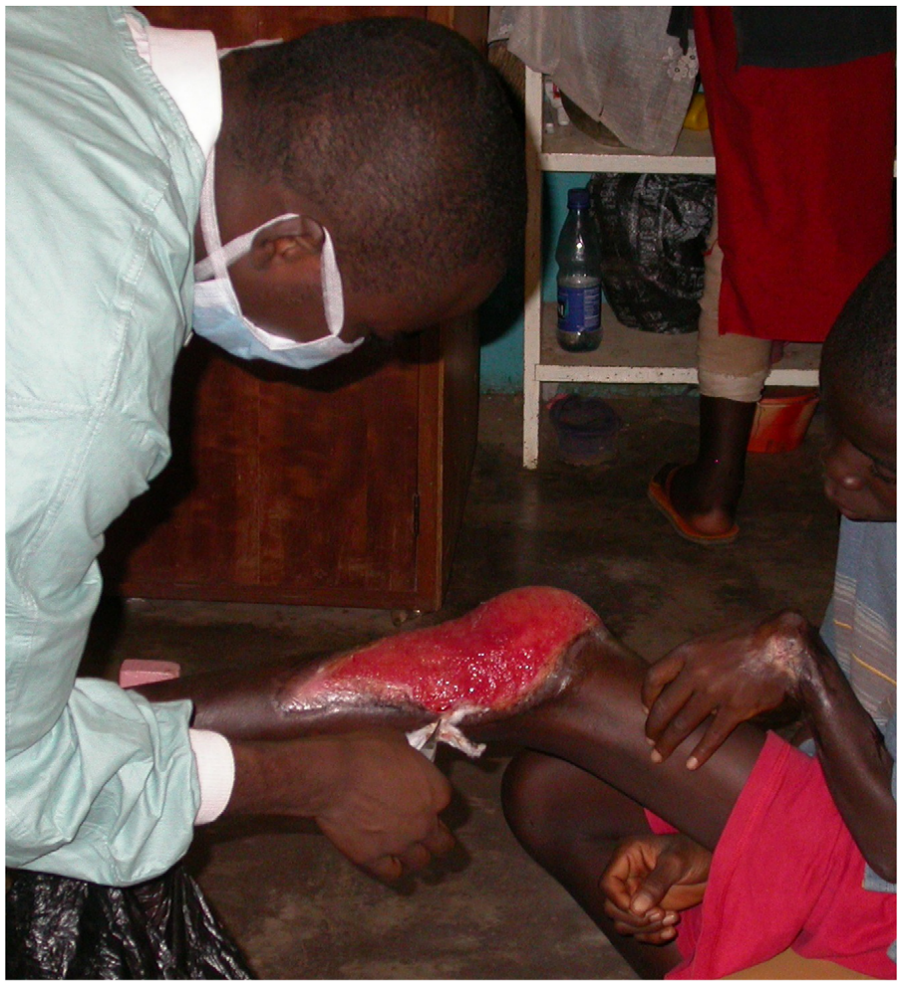
Buruli ulcer on leg and contractual deformity on wrist and hand. (Photo by R. Kimbirauskas).

Buruli ulcer (BU) is a serious necrotizing cutaneous infection caused by *Mycobacterium ulcerans* [1]–[7]. Before the causative agent was specifically identified, it was clinically given geographic designations such as Bairnsdale, Searles, and Kumasi ulcer, depending on the country [8]–[11]. BU is a neglected emerging disease that has recently been reported in some countries as the second most frequent mycobacterial disease in humans after tuberculosis (TB) [12]–[14]. Large lesions often result in scarring, contractual deformities, amputations, and disabilities [2]–[4], [7], [14]–[22] (Fig. 1). Approximately 80% of the ulcers are located on the limbs, most commonly on the lower extremities yet some variation exists [3], [13], [23], [24]. In Africa, all ages and sexes are affected, but most cases of the disease occur in children between the ages of 4–15 years [5], [13], [17], [25]–[28].

**Figure 2:**
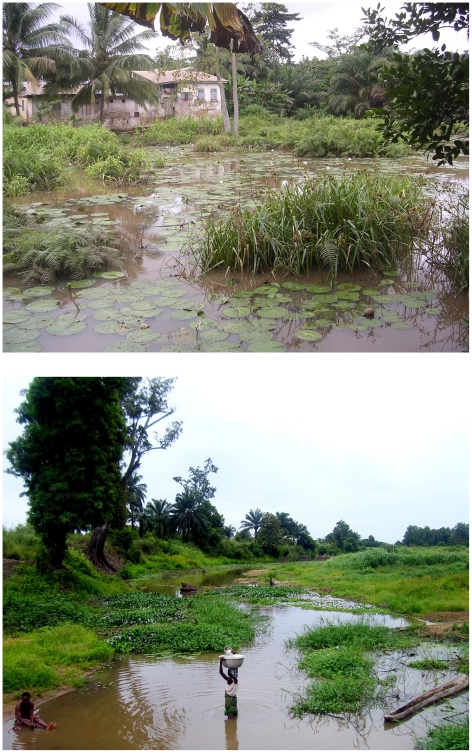
Typical Buruli ulcer riverine endemic sites in Ghana and Benin, respectively. (Photos by M. E. Benbow and M. McIntosh, respectively).

**Figure 3:**
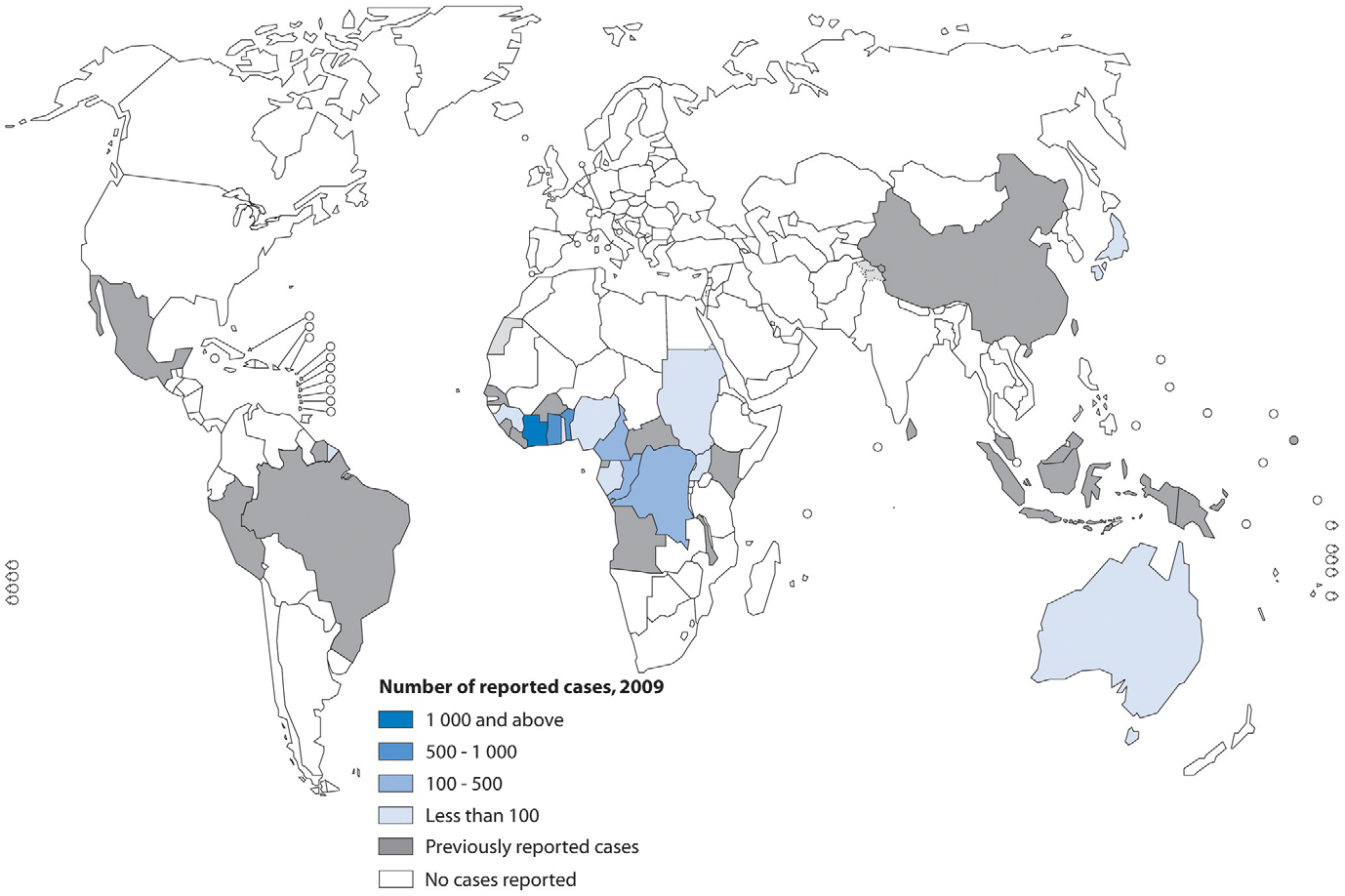
A global map representing countries that have reported cases of Buruli ulcer disease as of 2009 (WHO).

BU is a poorly understood disease that has emerged dramatically since the 1980's, reportedly coupled with rapid environmental change to the landscape including deforestation, eutrophication, dam construction, irrigation, farming (agricultural and aquaculture), mining, and habitat fragmentation [3]–[7], [29], [30]. BU is a disease found in rural areas located near wetlands (ponds, swamps, marshes, impoundments, backwaters) and slow-moving rivers, especially in areas prone to flooding [3], [4], [23], [27], [29], [31]–[36] (Fig. 2). Cases have been reported from at least 32 countries in Africa (mainly west), Australia, Southeast Asia, China, Central and South America, and the Western Pacific [3], [6], [20], [28], [37], [38] (Fig. 3). A number of cases have been reported in non-endemic areas of North America and Europe as a sequel to international travel [20], [39]–[42].

Buruli ulcer disease is often referred to as the “mysterious disease” because the mode of transmission remains unclear, although several hypotheses have been proposed. The objectives of this article are to: 1) review the current state of knowledge on the ecology and transmission of *M. ulcerans*, 2) discuss traditional and non-traditional methods for investigating transmission, and 3) suggest an intellectual framework for establishing criteria for transmission.

## Methods

### Data Sources and Search Strategy

Selection of the publications cited was based on the following approaches: 1) Direct knowledge of the authors of this manuscript regarding their background in the field of Buruli Ulcer research and knowledge of key papers and unpublished data; 2) Online search engines for Buruli Ulcer and *Mycobacterium ulcerans* (predominantly PubMed, ISI Web of Knowledge, Web of Science, Centers for Disease Control (CDC); 3) Knowledge in the field of Buruli Ulcer research in that three of the authors (Merritt, Small, Johnson) are on the WHO Technical Advisory Committee for Buruli Ulcer in Geneva, Switzerland; 4) Review of the following websites: Buruli ulcer disease maintained by WHO in Geneva, Switzerland (http://www.who.int/buruli/en), The Buruli Ulcer Disease Ecology Research Consortium (BUDERC) (https://www.msu.edu/~budiseco/index.html); and UBS Optimus Foundation (http://www.stopburuli.org).

## Results and Discussion

### The Pathogen

*M. ulcerans* is a slow-growing environmental mycobacterium that can be isolated from primary lesions after a 5–8 week incubation period, although up to 6 months may be required [43], [44]. *M. ulcerans* falls into a group of closely related mycobacterial pathogens which comprise the *M. marinum* complex. The *M. marinum* complex contains mycobacterial species pathogenic for aquatic vertebrates and includes *M. marinum* (fish), *M. pseudoschottsii* (fish) and *M. liflandii* (frogs) [45]–[48]. All of these species are characterized by slow growth rates and low optimal growth temperatures [49]. From a genomic standpoint, the species in the *M. marinum* complex can be considered a single species based on the fact that they share over 97% identity in the 16sRNA gene sequence [50]. However, practical considerations have led to the establishment of separate names based on differences in host tropism and pathogenesis analogous to other mycobacterial groupings, such as the *M. avium* and *M. tuberculosis* complexes.

Genomic analysis suggests that *M. ulcerans* evolved from an *M. marinum-*like ancestor [21], [51] through the acquisition of a large virulence plasmid and accumulation of multiple copies of insertion sequences, IS*2404* and IS*2606*. The genome has undergone considerable reductive evolution through a number of mutational events including transposon insertion. As a result, the genome has accumulated over 700 pseudogenes [21], [52]. Although it has been reported that micro-aerophilic conditions enhance the growth of *M. ulcerans* in the BACTEC system [53], the *M. ulcerans* genome strain lacks both nitrate and fumarate reductase systems, suggesting that *M. ulcerans* is considerably handicapped in the ability to grow under low oxygen conditions compared with *M. marinum*. The reported discrepancy in the oxygen requirements of *M. ulcerans* may be due to strain differences and requires closer investigation. A mutation in *crtI*, a key gene in the pathway for carotinoid biosynthesis, is suggested to compromise the ability of *M. ulcerans* to survive in direct sunlight [52]. A number of genes in ion transport and lipid biosynthesis have been lost and the repertoire of PE, PPE genes are considerably reduced compared with *M. tuberculosis* or *M. marinum*. Taken together, these results suggest that *M. ulcerans* is undergoing adaptation to a different and narrower niche than *M. marinum.* This idea has recently gained support from experimental work in which Medaka fish were infected with *M. marinum* and *M. ulcerans*. In these studies, *M. marinum* produced a lethal infection in Medaka, whereas *M. ulcerans* was not pathogenic and declined over a 23-week infection period (L. Mosi, unpubl. data).

The most important phenotypic characteristic of *M. ulcerans* is the low optimal growth temperature and the extremely restricted growth temperature range. *M. marinum* exhibits growth between 25–35°C, although the optimal growth temperature is 30–35°C [54], [55] and many *M. marinum* isolates are capable of growth at 37°C. In contrast, growth of *M. ulcerans* strains under laboratory conditions is characterized by a remarkably narrow temperature range between 28–34°C and optimal growth of most strains is found between 30–33°C [56]. The restricted growth temperature of *M. ulcerans* is thought to play a substantial role in the pathogenesis of BU by limiting infection to the skin. The organism has never been isolated from internal organs of human patients or from bone in cases of osteomylelitis, or from the internal organs or blood of experimentally infected animals [51], [57]–[59]. It has been recently reported that many isolates of *M. ulcerans* survive at 37°C for 13 days, although numbers decline after the first few days. No one has isolated or derived a strain capable of growth at 37°C [60].

The characteristic pathology of BU is mediated by a polyketide-derived macrolide exotoxin called mycolactone, which is cytotoxic and immunosuppressive [51], [61], [62]. Because of the large metabolic cost of producing mycolactone, it is likely that mycolactone plays an important role in the survival and growth of *M. ulcerans* in its environmental niche.

### Ecology and Distribution of the Pathogen and Disease

#### Detecting *M. ulcerans* in the environment

The slow growth rate of *M. ulcerans* and the complex mix of many faster growing bacteria and fungi in environmental samples have prevented direct culture on artificial media of *M. ulcerans* from the environment. A major breakthrough in environmental studies occurred with the development of the first PCR probes for *M. ulcerans* based on detection of IS*2404* by Ross et al. [63]. This technique was rapidly adopted by a number of laboratories leading to identification of *M. ulcerans* DNA in environmental samples including detritus, soil, biofilms, water filtrates, fish, frogs, snails, insects and other invertebrates [18], [35], [64]–[75].

Although IS*2404* PCR has become the gold standard for clinical diagnosis of Buruli ulcer, there are several caveats in applying these methods to environmental samples. First, PCR detects DNA, not intact organisms. The death of infected organisms will lead to the release of *M. ulcerans* DNA into the environment where it may stick to a number of substrates. Although in two different countries in Africa, Williamson et al. [67] found *M. ulcerans* DNA in 9.7% (8/82) of water filtrant samples and Vandelannoote et al. [59] found 7.7% (1/13) water samples positive for *M. ulcerans*, the significance of these small quantities of *M. ulcerans* in an environmental sample is difficult to evaluate. In southeastern Australia, *M. ulcerans* also has been detected in a range of environmental samples. Recently, Fyfe et al. [76], reported that 30% of selected samples including detritus, plant material, suspended solids, and soil collected from one highly-endemic area were weakly positive by quantitative PCR. However, in a low endemicity area, only 4/156 (3%) of samples (2 soil, 2 terrestrial plant) were positive. Interpretation of results from environmental PCR is complex. PCR methodology detects DNA, but it does not provide definitive proof for the presence of intact bacteria in a matrix. DNA bound to the surface of potential vectors in the water column also will be detected. However, the successful culture of *M. ulcerans* from an aquatic water bug collected in Benin [71] provides definitive evidence for the presence of *M. ulcerans* in an aquatic invertebrate. This considerable achievement was based on earlier observations using IS*2404* PCR that implicated aquatic water bugs as possible reservoirs or vectors of *M. ulcerans* [70].

#### Ecological associations with disturbed water bodies

Until recently, a systematic and/or quantitative approach to the ecology of *M. ulcerans* in the environment has received little attention, despite the fact that nearly all epidemiological studies have associated disease outbreaks with villages in close proximity to human-disturbed aquatic habitats, including both standing and moving water bodies [7], [9]–[11], [19], [20], [25], [33], [77]–[80]. Increased BU incidence has been reported in association with: 1) unprecedented flooding of lakes and rivers during heavy rainfall [9], [16], [30], [37], [81]; 2) the damming of streams and rivers to create impoundments and wetlands [4], [9], [30], [37]; 3) resorts that modify wetlands [16], [30]; 4) deforestation practices and increased agriculture leading to increased flooding [4], [9], [18], [30], [37]; 5) construction of agricultural irrigation systems [4], [30], [81]; 6) rice cultivation [4], [9]; 7); alluvial, pit and sand mining operations [30], [37], [82]; and 8) population expansion, resettlement and migration closer to water bodies [9], [16], [18], [27], [30], [37].

Indeed, many water bodies associated with increased sedimentation and eutrophication have low dissolved oxygen concentrations that may enhance the growth of *M. ulcerans* [53]. Hayman [9] speculated that in Australia *M. ulcerans* enters surface waters through deforestation, erosion and run-off contamination. He suggested that populations of *M. ulcerans* were washed into aquatic habitats where environmental conditions facilitated growth and proliferation, much like an algal bloom. Because most infectious diseases have a strong correlation between infective dose and incubation period for disease, Hayman [9] speculated that slow growth of *M. ulcerans* might be required for the bacteria to achieve population numbers sufficient to produce infection and the appearance of disease. The way in which *M. ulcerans* could be washed down into these habitats has never been explained, but is consistent with other reports of increased BU outbreaks associated with deforested and heavily flooded African lands [20], [33].

Further, deforestation leads to lost riparian cover, resulting in increased water temperatures that may facilitate *M. ulcerans* growth at optimal temperatures of 30–33°C [11], [18], [20]. Associated sedimentation (e.g., turbidity) also would provide ultraviolet light (UV) attenuation and protection for *M. ulcerans* biofilm near the bottom substrates and on submerged plant surfaces as proposed by Merritt et al. [30]. It has been documented that UV lowers *M. ulcerans* cell viability [52], and thus deforestation and high-impact agriculture may promote increased nutrients, higher temperatures, UV attenuation and lower dissolved oxygen – environmental conditions that facilitate *M. ulcerans* growth.

Because of the association with freshwater habitats, Eddyani et al. [83] hypothesized that freshwater plankton, specifically protozoans, may act as reservoirs for *M. ulcerans*, or may even facilitate the multiplication of the bacteria [18]. Although the former authors did not detect *M. ulcerans* DNA in free-living amoebae collected BU endemic areas in Benin, this area of research definitely warrants further investigation.

#### Landscape ecology of the disease

Buruli ulcer has been widely associated with proximity to aquatic habitats. The disease is rare in the savanna regions of West Africa and drier areas of Australia. Its presence in Australia is notably costal however, where water is often saline. This association between ecosystem ecology and disease has not been quantified. Rather, the association is most often anecdotal or related to specific human risk factors (e.g., wading, swimming, fishing, bathing, washing, farming, mining, etc.) in different countries and/or regional districts (see review below). To date, there have been few ecological studies focused on statistically determining why residence near certain water bodies is associated with BU, whereas the disease is absent along others [30], [67], [68]. For example, BU is highly associated with residence along several major river systems in both Benin and Ghana [12], [14], [20], [84], [85], whereas disease is essentially non-existent in communities within a few kilometers of Lake Volta, the largest water system in Ghana, as well as along the Mono River in Benin. Williamson et al. [67] recently found that in Ghana, PCR results suggesting that *M. ulcerans* and/or other mycolactone producing mycobacteria are widely distributed in water bodies in endemic and non-endemic villages. In these studies, however, the identification of endemic versus non-endemic sites was based on passive surveillance. A community was considered endemic if a case had been identified in the public health center in the past three years. A community that is not listed in the health center records, in association with a case of Buruli ulcer, was considered non-endemic. A preliminary survey to validate the non-endemic status of several communities in the GA district of Ghana through active surveillance showed that Buruli ulcer cases could be indentified in nearly all of the villages visited along the Densu River in the GA district (P. C. Small, unpubl. data). In areas where much of the disease is not reported, this can lead to significant error in the designation of “non-endemic.”

There have been case control studies and observational reports of disturbed landscape associations with BU disease [29], [30], [86]; however, there have only been a few recent studies to statistically quantify landscape characteristics and relationships with disease [36], [79], [81], [87]. Duker et al. [79] found that arsenic levels in soil and gold mining were significant covariates related to increased disease risk in the Amansie West district of Ghana, while Wagner et al. [36], [81] addressed larger scale land use/land cover relationships using satellite imagery, GIS, and country wide BU data from Benin. In the latter studies, Wagner et al. [36], [81] reported highest disease in communities surrounded by an agriculture matrix, and thus deforestation, with abundant wetlands and other habitats that experience frequent flooding. These were low-lying areas with complex topography far removed from urban settings [36], [81]. In another country-wide study using GIS, Brou et al. [88] found that in Côte d'Ivoire, communities near landscapes of irrigated rice and other agriculture near dams used for irrigation were related to increased risk of BU. These studies confirm previous epidemiological studies and indicate that there are quantifiable relationships between landscape features and land use that are related to BU disease. It is also clear that communities involved with these activities are at high risk for disease, yet how specific activities are associated with transmission remains unresolved.

#### Risk factors associated with Buruli ulcer disease

Recently, Jacobson and Padgett [89] systematically reviewed the risk factors associated with *M. ulcerans* infection throughout the world and concluded that poor wound care, failure to wear protective clothing, and living or working near water bodies were commonly identified risk factors in most studies. However, a number of epidemiological studies have identified other potential risk factors associated with *M. ulcerans* infection and these are summarized in Table 1. For each specific risk factor investigated, it is stated as to whether or not there was an increased or decreased risk of infection reported, or if the factor was not considered a risk factor in the analysis. Several of the commonly reported risk factors showed few consistent associations depending on the country, type of analysis conducted, use of different case definitions, and based on the control populations used [89]. For instance, in a case-control study from Ghana, Aiga et al. [25] found that swimming in rivers on a habitual basis was a significant risk factor, whereas drinking, cooking, washing clothing and bathing were not. However, in another Ghanaian study, wading, bathing, and swimming were all confirmed to be significant risk factors for BU [77]. Two studies found a decreased risk of infection with mosquito net use, while another study found no association between bed net use and infection (Table 1). However, in a case control study performed in southeastern Australia, use of insect repellent was associated with reduced risk and the reporting of mosquito bites on the forearms and lower legs was associated with increased risk [90]. Despite the association with water contact, fishermen were not found to be at high risk for the disease (Table 1). Although a review of these potential risk factors suggests that transmission of *M. ulcerans* might occur through direct inoculation of bacteria into the skin via contact with environmental sources, insect bites or trauma, it was clear that additional comparative studies are required to clarify the potential modes of transmission of *M. ulcerans* [89].

**Table 1:** A summary of reported risk factors associated with infection *Mycobacterium ulcerans*.

Country	Risk Factor(s)	Increased Risk of Infection	Decreased Risk of Infection	Not Considered a Risk Factor	Citation
Ghana	1) Arsenic-enriched drinking water (from mining)	X			Duker et al. (2004)
Ghana	1) Exposed skin2) Bednet and mosquito coils use3) Insect bites, cuts, scratches, and other wounds4) Exposure to riverine areas (wading and swimming)5) Association between BCG and vaccination or HIV infection6) Not wearing protective clothing7) Fishing	XXX		XXXX	Raghunathan et al. 2005
Ghana	1) Age 2–14 years of age2) Use of water for drinking, cooking, bathing, washing3) Association with agricultural activities4) Swimming in rivers	XX		XX	Aiga et al. 2004
Benin	1) 5–14 years of age2) Unprotected water from swamps3) BCG-vacinated patients >5 years old4) Participated in agricultural activities5) Sex	XXXX		X	Debacker et al. 2004, 2006
Benin	1) Mosquito bed net use2) Association with agricultural activities3) Improper wound care	X	X	X	Nackers et al. 2007
Cameroon	1) Living near cocoa plantation or woods2) Wading in swamps3) Wearing protective clothing while farming4) Association with agricultural activities5) Improper wound care6) Bed nets7) Mosquito coils8) Unprotected water sources9) Fishing	XXX	XX	XXXX	Pouillot et al. 2007
Cote d′ Ivoire	1) Age group2) Wearing protective clothing during farming activities3) Washing clothes4) Swimming5) Fishing		XX	XXX	Marston et al. 1995
Australia	1) Wearing protective clothing2) Use of insect repellent3) Most patients > 60 years old4) Washing wounds after sustaining minor skin trauma5) Exposure to mosquitoes	XX	XXX		Quek et al. 2007

Although there have been reports of a seasonal distribution in BU cases related to rainfall-influenced patterns of village waterbody usage [32], and by season in southeastern Australia [91], other studies have not shown this relationship [12]. Recording monthly trends for BU cases over a 3-year period in Benin, Sopoh et al. [12] found consistent average monthly BU case occurrence, without an apparent seasonal trend. However, country-wide data can obscure local variation in climate and the issue of seasonal trends needs to be more closely investigated at the local level. The unknown incubation period for Buruli ulcer, which may vary from 2 weeks to 7 months [92], [93], also makes it difficult to analyze seasonal factors with Buruli ulcer occurrence. Duker et al. [4], and more recently Marion et al. [94], discussed seasonal variations and *M. ulcerans* infections reported from different countries and concluded that there may be a temporal relationship between BU incidences and relatively dry periods; however, it also has been reported that *M. ulcerans* infections occurred mainly after flooding events [9], [16], [33], [34], [95].

### Environmental Reservoirs and Transmission

#### Africa

Unlike leprosy and tuberculosis, which are characterized by person-to-person transmission, it is hypothesized that *M. ulcerans* is acquired through environmental contact. Direct human to human transmission of *M. ulcerans* is extremely rare. The one reported case occurred following a human bite [96]. In this instance it was hypothesized that the patient's skin surface was contaminated with *M. ulcerans* from an environmental source (e.g. swamps) and driven into the skin by the playmate's bite. Non-human mammals and reptiles have been tested in the environment without positive findings [95], and several arthropods (i.e., bedbugs, black flies, mosquitoes) in Africa associated with vectoring other disease agents tested negative in early studies [18], [32]. However, few organisms of each taxonomic group were tested in these studies, and insect sampling methods were neither systematically employed nor standardized. Buruli ulcer cases in wild and domesticated animals in Africa have not been reported [97].

Portaels and colleagues [70] were first to suggest that aquatic bugs (Hemiptera) might be reservoirs of *M. ulcerans* in nature, and recently they described the first isolation in pure culture of *M. ulcerans* from a water strider (Hemiptera: Gerridae, *Gerris* sp.) from Benin [71]. A survey study [18] based on detection of *M. ulcerans* DNA in aquatic insects (Hemiptera, water bugs; Odonata, dragonfly larvae; Coleoptera, beetle larvae) collected from African BU endemic swamps confirmed their earlier findings, and suggested that small fish might also contain *M. ulcerans* [66], [98]–[100]. Marsollier et al. [64], [66], [98]–[100] conducted a series of laboratory studies and demonstrated that *M. ulcerans* could survive and show limited replication within the salivary glands of biting aquatic bugs (Naucoridae: *Naucoris cimicoides*). In their experimental model they demonstrated that *M. ulcerans* could be acquired from feeding on inoculated insect prey (a blow fly maggot), transmitted to mice via biting; and that the infected mice subsequently developed clinical BU [66]. Although there has been some controversy regarding the interpretation of this work [68], [101], [102] and subsequent follow-up studies on tracing the pathogen through the bug [103], [104], Marsollier and colleagues concluded that biting water bugs belonging to the families Naucoridae (creeping water bugs) and Belostomatidae (giant water bugs) could be considered reservoirs, and most importantly could serve as vectors in the transmission of *M. ulcerans* to humans in nature. More recently, Mosi et al. [101] investigated the ability of *M. ulcerans* to colonize aquatic bugs (Belostomatidae) collected from Africa. Using a natural infection model in which *M. ulcerans*-infected mosquito larvae served as prey that were then fed to the predacious bugs, Mosi and colleagues confirmed Marsollier's finding that infected belostomatid bugs could become infected with *M. ulcerans* via feeding. However, they concluded that transfer of bacteria through feeding was most likely to have occurred through contact with the heavily colonized raptorial arms and other external parts of the belostomatid, rather than through saliva or contact with other internal organs as originally reported [66]. Together, these experiments indeed support the hypothesis that predaceous aquatic insects may play an important role in maintaining *M. ulcerans* within food webs in the aquatic environment [1], [30], [68], [70] but, as detailed below, their role in actual transmission to humans remains unclear.

The role of other non-insect aquatic invertebrates as intermediate hosts or environmental reservoirs for *M. ulcerans* has been suggested by several authors [30], [66], [70], [73], [99], and recently confirmed in more field research [67], [68]. It was experimentally confirmed that aquatic snails could be transiently colonized by *M. ulcerans* after feeding on *M. ulcerans-*containing aquatic plant biofilms [64]. Aquatic plant extracts stimulated biofilm formation, and increased the uptake of labeled metabolites by *M. ulcerans* in laboratory experiments [65]. In the field, Kotlowski et al. [73] recorded *M. ulcerans* DNA in aquatic snails from endemic regions of Ghana and Benin, and other studies have found that average estimates of *M. ulcerans* increased by two orders of magnitude in detritus compared to water [72]. More recently, Marsollier et al. [104] described an extracellular matrix associated with the biofilm of *M. ulcerans* that may confer selective advantages to the mycobacteria in colonizing various microhabitats in the environment. Based on these studies and extensive environmental studies by Williamson et al. [67], it is evident that *M. ulcerans* DNA can be detected within biofilm on the plant surface, and as part of decaying organic matter (detritus) both of which serve as food for certain aquatic invertebrates and fish, suggesting reservoirs and movement throughout the aquatic food web.

A conceptual model, expanded and modified from Portaels et al. [70], illustrating the potential reservoirs and movement of *M. ulcerans* within and among aquatic environments was detailed by Merritt et al. [30] and more recently by Marion et al. [94]. Basically, *M. ulcerans* has been reported from mud, detritus, water filtrants, and plant biofilms, thereby allowing grazing or filtering aquatic insects (e.g., midges and mosquito larvae) or other invertebrates (snails, crustaceans, plankton) to concentrate mycobacteria through their feeding activities. Then, predatory aquatic vertebrates (i.e., some fish) and invertebrates (e.g., true bugs, beetles and dragonfly larvae) feed on other invertebrate prey or small fish, serving to move *M. ulcerans* from prey to biting insects. Lastly, aquatic insects capable of flight, and birds that prey on fish and/or aquatic invertebrates may potentially disseminate *M. ulcerans* to other aquatic environments [30].

Although the potential for different aquatic invertebrates in Africa to serve as environmental reservoirs for *M. ulcerans* has been clearly demonstrated, direct transmission by biting water bugs, other than by purely accidental means appears very unlikely for the following reasons. First, in Africa *M. ulcerans* DNA has only been detected in invertebrates that are not hematophagous. Predatory semi-aquatic Hemiptera (i.e., Naucoridae, Belostomatidae, Notonectidae) mainly feed on invertebrates (aquatic insects, Crustacea, snails) by inserting their piercing mouth parts into their prey, injecting saliva containing proteolytic enzymes, and then imbibing the liquefied prey tissues [105], [106]. Most employ an ambush strategy, waiting motionless clinging to vegetation for unsuspecting prey (Belostomatidae), while others may actively swim and pursue their prey (Naucoridae, Notonectidae) [107], [108]. Adults of most species of semi-aquatic Hemiptera possess the ability to disperse by flight, but mainly at night, and end up being attracted to electric lights during the breeding season, often correlated with the lunar cycle. Because of this, they often find their way into houses by accident [107], [108]. However, the very low disease prevalence among children less than three years of age suggests that infection does not occur in the house. When humans accidently come into contact with the bugs in the water, on aquatic vegetation, or away from water, they can be bitten [109]. However, these bugs do not actively search for humans, they do not require a blood meal or protein source to mature their eggs, nor is there any evolutionary history suggesting or supporting a vectorborne/pathogen transmission or co-evolving host/parasite relationship in the semi-aquatic Hemiptera [107], [110]. Therefore, based on the biology and behavior of predaceous aquatic insects, biting humans appears to be a rare event associated with a purely defensive reaction of these bugs [109], [111]. It should be noted, however, that the causative agent of Chagas disease (*Trypanosoma cruzi*) in humans is transmitted by a terrestrial hemipteran (Reduviidae), but it is through fecal contamination and not by the bite of the bug. Also, in this case the habitat of the vector (bug) is closely tied to that of its host [112].

In general, field studies on the prevalence of biting aquatic invertebrates do not support the hypothesis that biting aquatic bugs are vectors of *M. ulcerans* in nature; however, a recent study by Marion et al. [94] in Cameroon identified several water bug families as hosts of *M. ulcerans* in a Buruli ulcer endemic area. However, in Marion et al. [93], only one endemic area and one non-endemic area were evaluated, suggesting no replication, and thus, a limitation to testing how variable *M. ulcerans* is among endemic versus non-endemic areas/villages. This makes it difficult to compare to studies by Williamson et al. [67] and Benbow et al. [68] where multiple replicate sites were evaluated to test for *M. ulcerans* variability in standardized ecological samples. Benbow et al. [68] conducted the largest field study to date that examined biting water bugs in 15 disease-endemic and 12 non-disease-endemic areas of Ghana, Africa. From collections of over 22,000 invertebrates, they compared composition, abundance and invertebrate-associated *M. ulcerans* positivity among sites, and concluded that biting hemipterans were rare and represented a very small percentage of invertebrate communities. When endemic and non-endemic areas were compared, there were no significant differences in hemipteran abundance or invertebrate-*M.ulcerans* positivity rates (by PCR) between the areas, and there were no significant associations between hemipteran abundance and overall invertebrate-*M.ulcerans* positivity. Thus, there is little field evidence to support the assertion that biting bugs are major vectors of *M. ulcerans* in nature. However, as concluded by Marion et al. [94], the detection of *M. ulcerans* in water bugs in a specific area could possibly be used as an environmental indicator of the risk of *M. ulcerans* transmission to humans.

#### Australia

In Australia, infection with *M. ulcerans* occurs at low-levels in the wet tropical north where the climate is similar to sub-Saharan Africa [113]–[115]. However, more than 80% of Australia's cases of Buruli ulcer in the past 15 years have been in the temperate southeastern state of Victoria [93]. In comparison to Africa, people in Victoria have less direct contact with the environment, yet in two well-described outbreaks, 1.2–6.0% of the entire resident population in the outbreak areas developed Buruli ulcer [35], [116]. Visitors may also be at risk, and in one case, contact with an endemic town for just one day appeared to be sufficient to develop Buruli ulcer up to 7 months later [35].

In attempting to understand possible modes of transmission, two competing models have been proposed to explain this pattern of limited environmental contact, brief exposure, and high attack rates. Hayman [9] proposed that transmission by aerosol could partially explain outbreaks of *M. ulcerans* disease and an opportunity arose to test this hypothesis during a three year period when a large cluster of Buruli ulcer cases occurred in East Cowes, Phillip Island. This outbreak was significant in that only part of the town was affected, and there was a newly created wetland and a golf course at the center of the affected area. The golf course used a mixture of ground water and recycled water for irrigation and run-off from the golf course was likely to have drained towards the new wetland, connecting the two systems. Many of the case-patients lived close to the wetland or the golf course, supporting the concept of transmission by drifting aerosols from contaminated irrigation water [116]–[119].

Initially, no method existed for detection of *M. ulcerans* in environmental samples. However, as part of the outbreak investigation, Ross et al. [63] discovered IS*2404*, a high copy number insertion sequence in *M. ulcerans*. A PCR method using IS*2404 as* a target sequence has rapidly become the diagnostic method of choice for Buruli ulcer due to its high sensitivity, specificity, and its speed compared with traditional culture methods. *IS2404* PCR was then adapted for application to environmental samples, and positive results were obtained from the wetland and golf course irrigation system-the first direct evidence that *M. ulcerans* DNA is present in environmental samples.

*IS2404 PCR* also can be used as a preliminary test for the presence of *M. ulcerans* in Africa, but aquatic mycobacteria associated with disease in fish and West African clawed frogs (*Xenopus tropicalis*) also contain *IS2404*. For this reason, *IS2404* lacks sufficient specificity for use as sole criteria for *M. ulcerans* in Africa. To date, there is no evidence from Australia of the presence of *IS2404* in any other environmental mycobacterium.

The above findings supported the hypothesis that the golf course irrigation system and nearby wetland at Phillip Island had become contaminated with *M. ulcerans*, although transmission by aerosol itself was not directly assessed [72], [120]. Drainage of the wetland, reduction in recycled water use, cleaning of the irrigation equipment at the golf course, and subsequent separation of ground water from recycled water were collectively associated with fewer cases in the following years. Buruli ulcer linked to Phillip Island is now rare; however, disease activity in at least one other Victorian endemic area also declined over a similar time frame without a specific intervention, making it difficult to conclude that the environmental alterations made at Phillip Island were directly responsible for the decline in cases. During the same period several possums (Australian native tree-dwelling marsupials) with Buruli ulcer were identified at Phillip Island [18], the significance of which will be discussed further below.

In 2002, a new outbreak commenced in a small town on the Bellarine Peninsula about 60 km to the west of Phillip Island, also in coastal Victoria, southeastern Australia. More than 100 people who either live in or have visited Point Lonsdale have now been diagnosed with Buruli ulcer [35]. Several other towns on the Bellarine Peninsula have been linked to cases, but in lower numbers thus far. Although Point Lonsdale also has a golf course, it is not centrally located, and does not use recycled water. In 2004, intense local mosquito activity seemed to be associated in time with new cases of BU and Buruli lesions were observed on ankles and elbows, and on the back where gaps in clothing could allow access for mosquitoes. In one case, Buruli ulcer developed on the ear of a child who was only briefly present in the outbreak area. The child's mother suspected a mosquito bite as the initiating event [35].

These observations led to a series of studies aimed at assessing a possible role for mosquitoes in the transmission of *M. ulcerans*. Using an improved real-time quantitative IS*2404* PCR environmental screening method [74], more than 11,000 adult mosquitoes captured at Point Lonsdale were tested, and *M. ulcerans* DNA was identified in or on an estimated 4.3/1,000 mosquitoes. Most PCR positive mosquito pools were *Aedes camptorhynchus* (Thomson), the most common species on the Bellarine peninsula; however, *M. ulcerans* DNA also was detected in one or more pools of four other species [35]. PCR amplification and sequence analysis of one variable number tandem repeat (VNTR) locus confirmed that mosquitoes were carrying *M. ulcerans* DNA, indistinguishable from that of the human outbreak strain [74], [121].

A review of notifiable diseases in Victoria in the period 2002-8, demonstrated a statistically significant correlation between notifications of Buruli ulcer and Ross River Virus/Barmah Forest Virus infections (RRV/BFV) – both of which are transmitted by mosquitoes – but there was no correlation with any other non-mosquito borne notifiable disease [122].

A case-control study, conducted on the Bellarine Peninsula including Point Lonsdale, showed that the odds of being diagnosed with Buruli ulcer were at least halved in respondents who frequently used insect repellent, wore long trousers outdoors, and immediately washed minor skin wounds, and were at least doubled for those who received mosquito bites on the lower legs or lower arms. In a multivariate model, after adjusting for age and location, use of insect repellent and being bitten by mosquitoes on the lower legs were found to be independently associated with Buruli ulcer risk [90].

In laboratory experiments using a green fluorescent protein (GFP) labeled *M. ulcerans* mutant, in which GFP was linked to the mycolactone toxin polyketide synthase promoter, it was shown that when fed as a single pulse to live mosquito larvae, *M. ulcerans*-GFP was able to persist through 4 larval instars in the mouth parts and midgut of the insect. This was not observed with a closely related *M. marinum*-GFP mutant that did not produce mycolactone [123]. This permissive effect of mycolactone on allowing *M. ulcerans* to selectively colonize aquatic insects also was observed in experiments using aquatic water bugs [66], [100], [104]. However, other investigators found equal colonization with mycolactone negative and wild type strains [101], and this earlier selective effect was not observed in a study on *M. ulcerans* colonization of mosquitoes conducted by Wallace et al. [124].The latter study found a nearly 100% infection rate was obtained when wild type *M. ulcerans,* an isogenic mycolactone-negative *M. ulcerans,* and *M. marinum* (a non-toxin producing potential progenitor of *M. ulcerans)* were used to infect mosquito larva. These findings are in line with the fact that mosquito larvae do not discriminately feed on specific bacteria or other foods unless ingestion is mediated by particle size [125], [126]. Differences in experimental conditions and bacterial strains used may help to explain these conflicting findings.

Collectively, the above transmission research conducted in southeastern Australia lends support to mosquitoes as being a possible vector of the pathogen for Buruli Ulcer disease in this region of the country (see Bradford Hill guidelines for a critical assessment, below). More recently, it also has been discovered that that 38% of ringtail possums (*Pseudocheirus peregrinus* (Boddaert)) and 24% of brushtail possums (*Trichosurus vulpecula* Flannery) captured at Point Lonsdale had laboratory-confirmed *M. ulcerans* skin lesions and/or *M. ulcerans* PCR positive feces (Fyfe et al. [76]). The exact sequence of events linking mosquitoes, humans, contaminated possum excreta and infected possums has yet to be determined, but direct or indirect mosquito transmission from a possum reservoir presents a parallel model with aerosol transmission from contaminated environmental water sources. Neither the aerosol nor mosquito transmission hypothesis in temperate Australia is incompatible with transmission by direct contact with the environment or by other vectors not yet examined. Future research on the biological relationships within each model will help to resolve the relative probability and plausibility of either mode.

### Criteria for Establishing the Role of Insect Vectors of *M. ulcerans*

Stringent criteria exist in biomedical research for indicting the roles of living agents as biologically significant reservoirs and/or vectors of pathogens. The application of these criteria to the transmission of *M. ulcerans* presents a significant challenge. The above review reveals that various routes of transmission may occur, varying amongst epidemiological setting and geographic region, and that there may be some role for living agents as reservoirs and as vectors of *M. ulcerans*, in particular aquatic insects, adult mosquitoes or other biting arthropods. It is also clear that the exact mode of transmission, if indeed there is a single mode, remains unknown. We briefly discuss the process by which a vector is incriminated to the point of as much certainty as is possible, and then discuss the application of this process to indictment of insect vectors for transmission of *M. ulcerans*. If Buruli ulcer is a vectored disease, intervention might be designed to reduce the possibility of transmission since there are possibilities other than suppressing vector populations.

Vector incrimination traditionally involves satisfying a set of criteria analogous to Koch's postulates, summarized by Barnett [127] as follows: (1) the vector must be shown to acquire the pathogen from an identified source such as an infected vertebrate host or other reservoir, and thereafter become infected with the pathogen; (2) the vector must be shown convincingly to have close associations with infected hosts, including humans, in time and space; (3) individual vectors collected in endemic settings must repeatedly be found infected with the pathogen; and (4) efficient transmission to competent vertebrate hosts must be demonstrated experimentally, under well controlled conditions, by individual vectors, such as by bite or other means of direct contact. These criteria accommodate mechanical transmission if infection includes recovery of the pathogen from the vector's body, without making any assumptions about replication of the pathogen on or in the vector. Further, they do not preclude the possibility of parallel modes of transmission other than vectors. For example, the causative agent of plague, *Yersinia pestis*, has a flea vector and during sporadic outbreaks is transmitted by flea bites; but these bacteria also are transmitted during epidemics in aerosols generated by sneezing of pneumonically-infected humans or animals such as cats, which is probably the predominant mode of transmission in epidemics [128]. Similarly, human infection with the causative agent of tularemia, *Franciscella tularensis*, may occur through direct contact with contaminated water, by aerosols, by contact with blood or infected tissues of animals, or by bites of infected ticks, deer flies, or mosquitoes [129], [130]. The causative agent of Rift Valley fever, a *Phlebovirus* in the family Bunyaviridae, is transmitted amongst infected vertebrate reservoirs (mainly ungulates) by mosquitoes; however, many human infections occur upon exposure to infected animal blood at the time of slaughter, by aerosolization, as well as by mosquito bites [131]. Another useful illustration is that of *Chlamydia trachomatis*, the causative agent of trachoma, where the transmission to human eyes has been definitively associated with contact by *Musca sorbens* flies (Diptera: Muscidae) that breed in human feces in various parts of Africa [132]. Despite this observation, other mechanisms of transmission for this disease are known, such as person-to-person contact with contaminated fingers and wash towels [133], [134]. In two of the above examples (plague and Rift Valley fever), the pathogen has a close biological relationship with, and dependency upon, insect vectors; neither pathogen could persist in nature without infecting their respective vectors. For tularemia and trachoma, vectors are not essential to pathogen persistence in nature, even though fly control in the latter case was shown to reduce incidence of disease in humans [135]. However, it is unlikely in the case of tularemia and trachoma that even highly effective fly control could eliminate human infection in endemic areas owing to other modes of transmission [133]. Therefore, using a critical approach to address the issue of insect vector incrimination for *M. ulcerans*, one must be cognizant of the relative biological dependency of this bacterium on an insect vector, and the potential for facultative and facilitative relationships between these bacteria and various insect “hosts” to exist which may be ancillary or even spurious to the essential and normal transmission modes.

The most thorough examination of the role of an insect vector for transmission of *M. ulcerans* stems from investigations of aquatic, predaceous Hemiptera (true bugs) as reviewed above, which go far in addressing and meeting Barnett's criteria. It is important to recognize that the vast number of studies of *M. ulcerans* in environmental samples provide qualitative, indirect evidence of *M. ulcerans* based on very sensitive methods for detecting *M. ulcerans* DNA. Such studies revealed repeatedly that natural infection by *M. ulcerans* in field-collected bugs occurred, but it was tempered by detection of *M. ulcerans* in many other aquatic insects [18], [67]. Thus, definitive incrimination of a single species or group of closely-related aquatic and semi-aquatic Hemiptera to the exclusion of other insects was not initially established. Other studies suggested natural contamination of the surfaces of these insects with *M. ulcerans* and suggested that *M. ulcerans* growth could occur as biofilms on the external appendages of such ‘bugs’ [101]. Thus, although aquatic and semi-aquatic Hemiptera and other insects found to harbor *M. ulcerans* in nature might provide habitat for the bacteria, along with numerous other living and non-living surfaces where biofilms could form [104], this is insufficient evidence for indicating an obligatory or even facultative vectorial role to these insects. Although the experiments reported by Marsollier et al. [64], [66], [98]–[100] suggested modest bacterial replication in internal tissues of bugs, acquisition of bacterial infection from a live source (infected fly maggots meant to simulate an infected prey item), and transmission to mice, this evidence does not establish natural infection coupled with transmission to humans. Finally, there has been no epidemiological association established between spatial and temporal distribution of contacts with aquatic Hemiptera, or bites by them, and development of Buruli ulcer in humans [68]. As reviewed above, the common understanding of the feeding habitats of aquatic and semi-aquatic Hemiptera does not include feeding on humans. More likely, infection in aquatic insects is associated with exposure to *M. ulcerans* in detritus and on biofilms formed on submerged materials, leading to a generalized distribution of *M. ulcerans* and *M. ulcerans* DNA in aquatic environments. In this particular scenario, despite the body of research on the topic, Barnett's criteria have not yet been fulfilled satisfactorily.

The recent research by Wallace et al. [124], whilst firmly documenting growth of *M. ulcerans* in mosquito larvae and transtadial infection after the molt, showed that infection did not persist upon metamorphosis to the adult stage. Thus, the link between presence of *M. ulcerans* in aquatic environments in which larval mosquitoes are found and adult mosquito infection with *M. ulcerans*, was not confirmed experimentally. However, these studies did show that *M. ulcerans* DNA could be detected on surface components of some adult mosquitoes. This brings up an important issue regarding experimental design and suggests that interpretation of PCR results obtained from whole insect lysates must be cautiously interpreted. These findings suggest that further research is required to confirm the association between mosquito bites, adult mosquito infection, and incidence of Buruli ulcer in humans in Australia (reviewed above), where a link between mosquito feeding on infected possums and transmission of the agent via the same species of mosquitoes was proposed (Fyfe et al.[76]). An analysis of blood host choice by mosquitoes, documenting blood feeding on both possums and humans in the area where human cases of Buruli ulcer are occurring, would be required as one element of satisfying Barnett's criterion #2. At best, Barnett's criteria for vector incrimination have not been completely satisfied for a mosquito vector role, but more compelling data may be forthcoming on this matter in the future.

A second approach to vector incrimination involves application of the Bradford Hill guidelines for establishing causation of infection and disease in epidemiological/ecological contexts [136]. Rather than rely upon experimental evidence, the Bradford Hill guidelines emphasize epidemiological/ecological association and use of logical inference to build up support and evidence for a strong conclusion of cause and effect, where A represents the “cause” and B the “effect” in the relationships under study [137]. The result is an “evidence hierarchy” that can be used in formal deduction [138], and represents an interdisciplinary approach to causal investigation in disease ecology. Here, “A” would be contact between an insect vector infected with *M. ulcerans*, and “B” would be human infection with *M. ulcerans*. The guidelines are qualitative in nature and do not require the clear endpoints of Barnett's criteria, yet represent a logical approach to the problem of cause and effect under epidemiological circumstances [139]. They are as follows (Table 2):

**Table 2:** Listing of Hill's guidelines (Bradford Hill guidelines, Hill 1965) for associating a role of insect vectors of pathogens causing human disease.

Term	Descriptor/Qualifier
1. Plausibility	Plausible, rational given knowledge of the biology of the putative vector, biology of the pathogen, and epidemiology of the disease. Specious associations would contraindicate a positive association.
2. Temporality	The insect vector must show a temporal association with infection in humans; in particular, infected vectors should be found in endemic areas immediately before human cases occur.
3. Strength	The association of the putative insect vector with human cases must be strong in time and space and in an epidemiological context. Correlation analysis supports the conclusion of strength if the correlation is positive.
4. Biological Gradient	Prevalence of human cases should co-vary with prevalence of infection in the insect population.
5. Consistency	Confirmed human cases should consistently be associated with infected insect vectors in time and space.
6. Alternate Explanations	Explanations other than those related to a role of an insect vector should be considered and ruled out, or validated.
7. Experimentation	Role of an insect species as a vector should be validated through experimental analysis with adequate controls and with realism in experimental design.
8. Specificity	Infection with *M. ulcerans* in humans occurs when, and only when, a bite by an infected insect occurs first.
9. Coherence	The association of human infection with insect transmission must cohere to knowledge of similar relationships in other similar associations.

(1) Plausibility. The cause and effect association of A and B must be plausible, that is, rational and lacking in speciousness. By this is meant that the association reflects the common understanding of the normal behavior and other attributes of both A and B, bringing the appropriate factors together in such a way that abnormally implausible (i.e., irrational) explanations must be discounted. In formal philosophy, plausibility must be demonstrated by sets of binary outcomes whose relationships are clearly defined propositions which can be resolved by the application of logical discourse [140]. Although plausibility can be formulated axiomatically, it cannot be analyzed statistically. It is important, therefore, not to confuse “plausible” with “probable” as the latter allows for rare and unusual circumstances and events to be explanatory under the right circumstances, whereas the former involves a rigorous, but non-probabilistic analytical process. Put more simply, plausibility addresses qualitatively how likely or unlikely it is that A results in B. A common problem in epidemiological scenarios that confronts plausibility is the issue of clusters of cases of infection (e.g., [134]), which may or may not have spatial associations with other nearby cases or with the landscape qualities near those cases [136]. In the case of Buruli ulcer and vector transmission of *M. ulcerans*, it is not implausible that Hemiptera and human cases are associated in time and space, but it is not plausible that there is a direct, causal relationship between the pair except in rare, accidental circumstances. Hence, there is insufficient evidence to conclude that biting hemipterans are a significant vector of *M. ulcerans*, although they may act as environmental reservoirs.

(2) Temporality. If A results in B, then A must consistently precede B in temporal sequence. For Buruli ulcer, there is no evidence that bites of particular insects consistently precede development of patent *M. ulcerans* infection in humans, although there is evidence that mosquito bites are associated with increased risk [90]. The problem with this guideline is the prolonged period of time between exposure and development of symptoms in Buruli ulcer disease. However, if bites from true bugs always preceded disease, patients are likely to remember these due to the painful nature of a naucorid or belostomatid bite, in contrast to bites by mosquitoes that often go unnoticed.

(3) Strength. Is the “strength” of the association great? For example, is there a statistically significant correlation between A and B in space and or time? The association between contact with water sources and *M. ulcerans* infection in humans is reasonably strong, but between insect bites and infection it is not for hemipterans, nor yet firmly established for mosquitoes in Australia and virtually non-existent for mosquitoes in Africa.

(4) Biological gradient or dose-response relationship. Infection in B should increase proportionately as A increases. This principle can operate at the dose-response level, as in a toxicological series; or at the population level, as when, e.g., more dengue virus infected mosquitoes results in more human cases of infection with that virus in space and time. The relationship may not be linear, thus confounding the interpretation of the relationship. There is no evidence that higher infection rate of *M. ulcerans* in aquatic insects results in higher incidence of infection in humans, although there is evidence that adult mosquitoes caught in highly endemic area in southeastern Australia are more likely to be PCR positive than those caught in areas with lower endemicity [35].

(5) Consistency. Episodes and research data where A and B show spatial and temporal associations commensurate with the other Bradford Hill guidelines must consistently reveal the association to be a positive one. Consistency could be revealed by meta-analysis of many data sets or through replicated, longitudinal studies across time and space. If scenarios emerge in which B occurs, but A does not in space and time, then doubt emerges regarding the veracity of the association. Although there are vignettes, correlations, and observations regarding insect vectors of *M. ulcerans*, there is no clear consistency among epidemiological scenarios to currently support the notion that insects are the predominant vector in most geographic regions. Consistent data are lacking for the ubiquitous role of vectors in the *M. ulcerans* transmission system.

(6) Consideration of alternate explanations and analogous situations. Explanations other than causation due to A must be carefully weighed as alternatives. Causation may be inferred by analogous correspondence with other scenarios. For Buruli ulcer, a wide range of alternate explanations for transmission exists, such as human behavior linkages involving activities that increase direct skin contacts with contaminated water and inoculation with infective doses of *M. ulcerans* through lesions. However, as we have seen, several diseases with insect vector associations have alternative transmission modes, such as tularemia, plague, Rift Valley fever, and trachoma. Thus, it is plausible that there are multiple modes of transmission in Buruli ulcer, with certain modes more likely given specific environmental and socio-cultural contexts.

(7) Experimentation. If experimental manipulations are feasible and can be structured realistically, then outcomes of the treatment regime conferred upon B (such as exposure to the effects of A) must reflect the association in a positive way. Often, however, Bradford Hill guidelines are utilized because experiments are either not possible, or not sufficiently rigorous or realistic. Experimental data on insect-*M. ulcerans* relationships have been reviewed above. There seems to be a sufficient body of work with sufficient variation in outcomes that the treatment manipulations do not lead to easily generalized conclusions on the association. Furthermore, it is often difficult to find true replication for large-scale experiments (e.g., treating replicate ponds with a specific chemical agent to test of changes in *M. ulcerans*), making it difficult to rigorously evaluate and experimentally test complex dynamics related to multiple modes of transmission of *M. ulcerans* within the environment.

(8) Specificity. In this guideline, B follows A, but B does not follow when other plausible explanatory factors and events occur in temporal or spatial association. It is one of the most difficult of the guidelines to satisfy and comes closest to a strict criterion, usually because of incomplete information, multiple causes of B, random effects, and systematic errors of measurement. The review of the literature on cause and effect between insects and Buruli ulcer cases indicates a paucity of data to prove specificity. Furthermore, there are few studies relating disease incidence and insect abundance in time and space especially in Africa, and none of the alternate explanations for transmission reviewed above, such as through aerosols (9), have been discounted. The current available data points to a multiple transmission model for Buruli ulcer, indicating that the Buruli ulcer disease system lacks specificity with regard to vector insects, with the possible exception of southeastern Australia. Therefore, more complete and rigorous qualitative assessments of data are critical to provide evidence for consistency and specificity with regard to the role of vectors and reservoirs in transmission of *M. ulcerans*.

(9) Coherence. The association of B with A must cohere to knowledge of similar relationships in other similar associations. For *M. ulcerans*, insect transmission is quite unusual, as the remainder of the *M. marinum* group does not depend upon invertebrate vectors for transmission and infection in fish hosts. Furthermore, there is no scientific precedent for transmission of any disease agent from the direct bites of hemipteran bugs, nor is there precedent for biological transmission of any bacterial pathogen by mosquitoes known. Thus, coherence is overall not strong. However, although closely related to *M. marinum*, *M. ulcerans* is a distinct species with a genomic signature indicating it has diverged from its free-living ancestor and now occupies a specialized niche environment. Either a vertebrate gastrointestinal tract (e.g. possums) or insects may provide this unknown microenvironment.

In summary, neither the application of Barnett's strict criteria nor the Bradford Hill guidelines support conclusively that bites by *M. ulcerans*-infected insects' result in human infection with *M. ulcerans*. However, further research will reveal if any associations might result in higher risk of infection under certain circumstances. Infection with anthrax bacteria, *Bacillus anthracis*, provides a useful comparison, not as a directly transferable model, but rather as a model for conceptualization of how insects, like mosquitoes, may have ancillary roles in bacterial transmission when other transmission modes also exist [141]. In that system, infection occurs in animals endemically and sporadically. When they are stressed (as in a drought), they become susceptible to low dosages of bacterial spores in soil. As animals die, colonization of necrophilic flies during decomposition results in infection locally and increased bacterial sporulation and more animal cases occur as a result (the so-called “case multipliers” effect of insects). As more animals become infected, an insect-mediated dispersal of bacteria occurs by biting flies such as deer flies and horse flies, whose mouthparts can become contaminated with bacteria during blood feeding (the so-called “space multiplier” effect of insects). The role of flies in both modes furthers epizootics of anthrax. Although these two processes are unlikely to occur for Buruli ulcer, which appears to be mainly an endemic disease, the scenario for anthrax establishes a model by which insects might be envisioned to have ancillary roles in transmission for *M. ulcerans* as well.

### Conclusions

#### Recommended research directions on Buruli ulcer disease

As stated in the beginning of this review, Buruli ulcer disease has been referred to as the “mysterious disease” because the exact mode(s) of transmission, in the strictest sense, remain unclear, although several hypotheses have been proposed. We have reviewed the hypotheses and reported on studies that provide good evidence of probable reservoirs for the disease, particularly in Australia. An intellectual framework for establishing criteria for transmission followed this. Finally, we recommend that the following research studies be conducted to help better understand transmission of *M. ulcerans* in nature: 1) in depth studies of human behavior patterns in African endemic villages to better understand exposure to the pathogen in the environment; 2) a search for mammalian and/or other animal reservoirs and potential arthropod vectors in Africa; 3) understanding the relationship between mosquitoes, humans and infected possums who frequently share the same habitats in Australia; 4) laboratory competency studies with Australian mosquitoes using local strains of MU to determine whether transmission could occur vertically (larvae to adult) or horizontally (adult feeds on possum and then on humans); 5) further field and laboratory experiments on vector transmission and vector competence to confirm current hypotheses and experimental evidence on arthropod transmission; and 6) the development of new and innovative studies aimed at satisfying Hill's Criteria to provide strong and logically defendable evidence about the true mode, or modes, of Buruli ulcer transmission in nature.

## Supporting Information

Checklist S1 PRISMA checklist.
(0.07 MB DOC)
